# The gut-lung axis: the impact of the gut mycobiome on pulmonary diseases and infections

**DOI:** 10.1093/oxfimm/iqae008

**Published:** 2024-07-24

**Authors:** Emily A Sey, Adilia Warris

**Affiliations:** Medical Research Council Centre for Medical Mycology, University of Exeter, Exeter, EX4 4QD, UK; Medical Research Council Centre for Medical Mycology, University of Exeter, Exeter, EX4 4QD, UK

**Keywords:** Mycobiome, gut-lung axis, lung infections

## Abstract

The gastrointestinal tract contains a diverse microbiome consisting of bacteria, fungi, viruses and archaea. Although these microbes usually reside as commensal organisms, it is now well established that higher abundance of specific bacterial or fungal species, or loss of diversity in the microbiome can significantly affect development, progression and outcomes in disease. Studies have mainly focused on the effects of bacteria, however, the impact of other microbes, such as fungi, has received increased attention in the last few years. Fungi only represent around 0.1% of the total gut microbial population. However, key fungal taxa such as *Candida*, *Aspergillus* and *Wallemia* have been shown to significantly impact health and disease. The composition of the gut mycobiome has been shown to affect immunity at distal sites, such as the heart, lung, brain, pancreas, and liver. In the case of the lung this phenomenon is referred to as the ‘gut-lung axis’. Recent studies have begun to explore and unveil the relationship between gut fungi and lung immunity in diseases such as asthma and lung cancer, and lung infections caused by viruses, bacteria and fungi. In this review we will summarize the current, rapidly growing, literature describing the impact of the gut mycobiome on respiratory disease and infection.

## Graphical Abstract

**Graphical Abstract:**
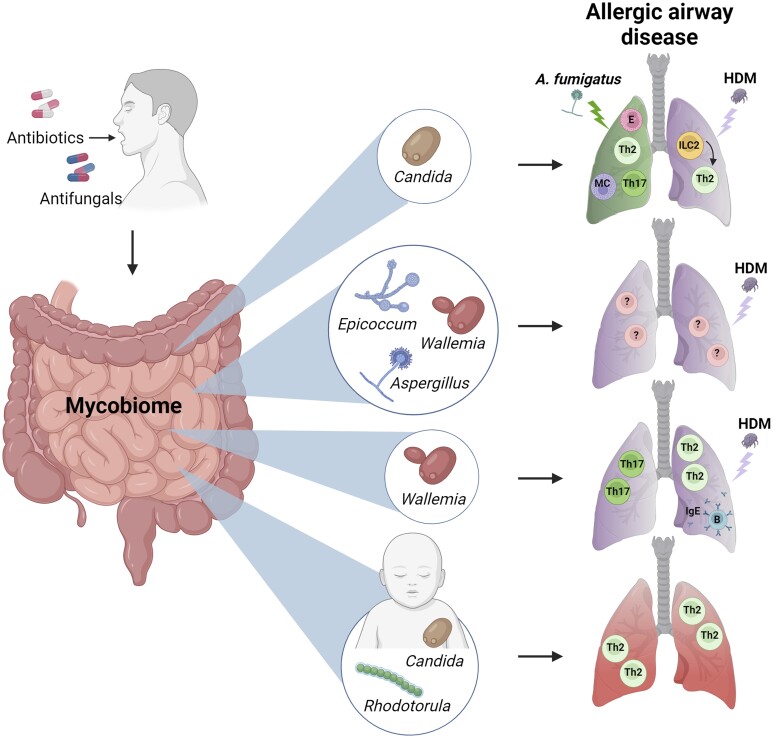
The gut-lung axis: the impact of the gut mycobiome on pulmonary diseases and infections. Fungal constituents of the gut microbiome, known as the mycobiome, can influence disease in the lung. Additionally, patients with lung disease have altered mycobiome compared to healthy individuals. This bidirectional communication is known as the gut-lung axis. Gut fungi have been shown to impact development, progression and outcome of diseases including asthma and lung cancer, viral infections including influenza and COVID-19, bacterial infections such as tuberculosis, and fungal infections including aspergillosis and histoplasmosis

## Introduction

The gastrointestinal (GI) tract is home to a multitude of microbial organisms, including bacteria, fungi, viruses, and archaea, collectively known as the microbiome. Bacteria are the dominant member of this community, while fungi compromise only around 0.1% of the total gut microbial population [1]. Despite their low abundance, recent advances in culture techniques and deep sequencing have revealed the substantial impact of these fungal constituents on intestinal homeostasis and disease [2–5]. This rapidly growing field of research has uncovered a diverse range of fungal species within the human gut, including key taxa, such as *Candida* species, which reside in the gut as commensals [6]. These discoveries are largely due to advances in deep sequencing technologies and the establishment of several databases dedicated to fungal species, such as FungiDB and Mycobank [7, 8]. The interaction of gut fungi with complex immune systems is aimed to maintain homeostasis and to prevent invasion and dissemination. The gut mucosa, acting as an anatomical barrier, serves to prevent the translocation of both commensal and pathogenic organisms into the bloodstream and distal organs [9]. Additionally, the gut has a significant population of immune cells that travel through the lymphatic system and regulate commensal host-microbe interactions by developing the host’s innate and adaptive immunity. The later includes mediating the balance of Th17/Treg immunity, which is important for control and resolution of fungal infections, and Th2 immunity which is involved in allergic responses, such as asthma [10].

The evolution and maturation of the gut mycobiome throughout life has been demonstrated to significantly impact health, and exhibits distinctive compositions in different age groups. In early life the human gut mycobiome mainly consists of *Saccharomycetales* and *Malasseziales*, until the shift from breast milk to solid food when *Saccharomyces cerevisiae* and *Candida* become the most abundant species [11, 12]. At the same time the appearance of other species such as *Cystofilobasidium* spp, species in Ascomycota phylum such as *Debaryomyces hansenii* and *Monographella* spp begin to appear [12, 13]. Over the human life span, the mycobiome appears to be more unstable than the bacteriome [14]. In adulthood, fungal diversity increases, including species from the phyla Ascomycota, Basidiomycota, and Zygomycota. More specifically, the healthy human gut shows a high abundance of yeasts including *Saccharomyces cerevisiae*, *Malassezia restricta*, and *Candida albicans*, and filamentous fungi such as *Penicillium*, *Cladosporium* and *Aspergillus* spp. [13–15]. Then in later life, after the age of 50 years, *Penicillium*, *Candida*, *Saccharomyces*, and *Aspergillus* are the most common genera [16]. Thus, age has a significant impact on the gut mycobiome composition, and relative abundance of these species in early life has been linked to development of various lung diseases in later life, such as asthma (discussed in detail below) [17].

Fungi contain highly immunologically reactive elements within their cell wall, including β-glucans, chitin, and mannose-associated complexes, that are recognized by various pattern recognition receptors on immune cells, including C-type lectin receptors and toll-like receptors [18]. These interactions trigger signal transduction pathways that drive antifungal immunity and maintain a balance to prevent fungal overgrowth and dissemination. In general, innate immune responses, e.g. phagocytosis by neutrophils and macrophages, clear invading pathogens in a non-specific manner. Antigen presenting cells, including macrophages and dendritic cells also trigger the adaptive immune cells including Th17 cells producing IL-17 and IL-22, and Th1 cells producing IFN-y, promoting further recruitment of neutrophils that prevent fungal overgrowth [19]. However, during chronic inflammation neutrophils are persistently recruited to the site of infection by cytokines such as IL-17, which is known to exacerbate disease in distant organs such as pulmonary fibrosis [20, 21]. Therefore, there is a fine balance between beneficial and detrimental cytokine responses during infection, particularly in patients with underlying disease. Importantly, gut derived macrophages and dendritic cells have the unique ability to maintain tolerance to gut commensals while preventing invasion and infection [22]. Germ-free (GF) animals, with no microbiome since birth, show altered abundance of innate immune cells. For instance, GF mice and pigs show reduced numbers of dendritic cells in the gut, while GF pigs have decreased systemic macrophages and GF rats are neutropenic [23–26]. Therefore, the microbiome plays an important role in shaping the numbers and composition of immune cells and host immune response.

Most of the research surrounding the impact of the gut mycobiome on disease focuses on gut diseases such as irritable bowel disease, coeliac disease, irritable bowel syndrome, colorectal cancer and *Clostridium difficile* infection, which have been reviewed elsewhere [27]. However, in the last decade interesting findings have linked the gut mycobiome to disorders beyond the gut. For instance, the influence of the gut mycobiome extends to distal sites, such as the heart, lung, brain, pancreas, and liver [28–32]. Presence of specific fungi within the diverse fungal milieu, has been linked to a spectrum of diseases ranging from autoimmune, metabolic, and neurological disorders to various cancers [12, 33–35]. Furthermore, disease within these distal organs is known to impact the composition of the gut mycobiome, emphasizing the concept of bidirectional communication [27]. In the case of the lung this phenomenon is known as the ‘gut-lung axis’. Although the focus has predominantly centred on the impact of bacteria, more recent research has revealed a growing body of evidence suggesting a substantive role for gut fungi within this inter-organ communication network.

While the exploration of the interactions between gut fungi and the immune system within the lung is in its infancy, early investigations have centred on a few diseases and infections. This evolving body of evidence highlights a substantial link between the gut mycobiome and lung health, particularly in the context of asthma and lung cancer, but also on pulmonary infections caused by viruses, bacteria and fungi. In this review we will summarize the current literature representing the gut mycobiome effects on respiratory diseases and infections.

## Gut mycobiome and lung disease

Lung diseases encompass a group of conditions affecting the airways and structures of the lungs, leading to persistent breathing difficulties. These diseases can significantly impact an individual's quality of life and include lung cancer, chronic obstructive pulmonary disease (COPD), asthma, interstitial lung diseases, bronchiectasis, and cystic fibrosis (CF) among others [36]. Recently studies have directly linked the development and progression of lung disease to relative abundance of specific species within the gut mycobiome. These studies largely focus on asthma and lung cancer with a couple of findings exploring the fungal mycobiome in other lung diseases.

### Asthma

Asthma is a chronic respiratory disease (CRD) and one of the most common immune-mediated disorders in the world. The latest statistics on worldwide prevalence of asthma from 2019 highlight its widespread impact, with over 250 million individuals experiencing asthma-related symptoms, and over 450,000 asthma related deaths annually [37]. The predominant form of asthma involves an allergic response entailing Th2 cells, eosinophils, and IgE antibodies, and in severe cases, Th17 responses are also implicated [38, 39]. Furthermore, various innate and adaptive immune cells, including dendritic cells (DCs), mast cells, basophils, innate lymphoid cells (ILCs), and Th9 cells, are associated with asthma [40]. Numerous factors contribute to asthma development including genetic predisposition, environmental factors, such as pollution, indoor mould growth and tobacco smoke, and alterations in the relative abundance of specific species within the gut microbiome [41, 42].

Over the past decade, research has increasingly linked the gut microbiome to asthma. In mice, asthma is commonly studied using sensitization models, that use common allergens such as house dust mite (HDM), or ovalbumin (OVA) [43]. These models mimic acute asthma symptoms such as excessive mucus production, airway hyperresponsiveness, and eosinophilic airway inflammation [10]. To assess a link between asthma and the microbiome, these experimental models of allergic airway inflammation have been utilized in GF animals, or mice treated with antibiotics. These mice showed exaggerated airway hyperresponsiveness and Th2-mediated inflammation compared to specific pathogen free (SPF) mice [44, 45]. Furthermore, administration of faecal microbiota from asthma patients to GF mice resulted in elevated oxidative stress and enhanced Th17 responses following OVA challenge [46]. Thus, linking the gut microbiota to increased hyperinflammation in the lung and asthma responses. Most studies exploring microbe involvement in these responses have focused on the bacterial components of the microbiome. These investigations demonstrate that the relative abundance of specific bacterial species, such as low levels of *Bifidobacterium*, *Lactobacillus*, and high levels of *Clostridium* in infants, may increase the risk of asthma development [47]. The fungal aspect of the microbiome has historically been understudied. However, the fungal component of the gut microbiome is now appreciated to show variation in asthma patients, and subsequently impacts disease development and progression. For instance, individuals with asthma demonstrate lower fungal richness, evenness, and diversity [48]. Furthermore, overgrowth of specific fungal species, such as *Candida* spp in gut have been linked to more severe asthma symptoms, and presence of specific immune cells, including Th2 cells, Th17 cells, eosinophils, mast cells, macrophages and ILC2 in the lung have been associated with these effects [48–51].

**Figure 1.**
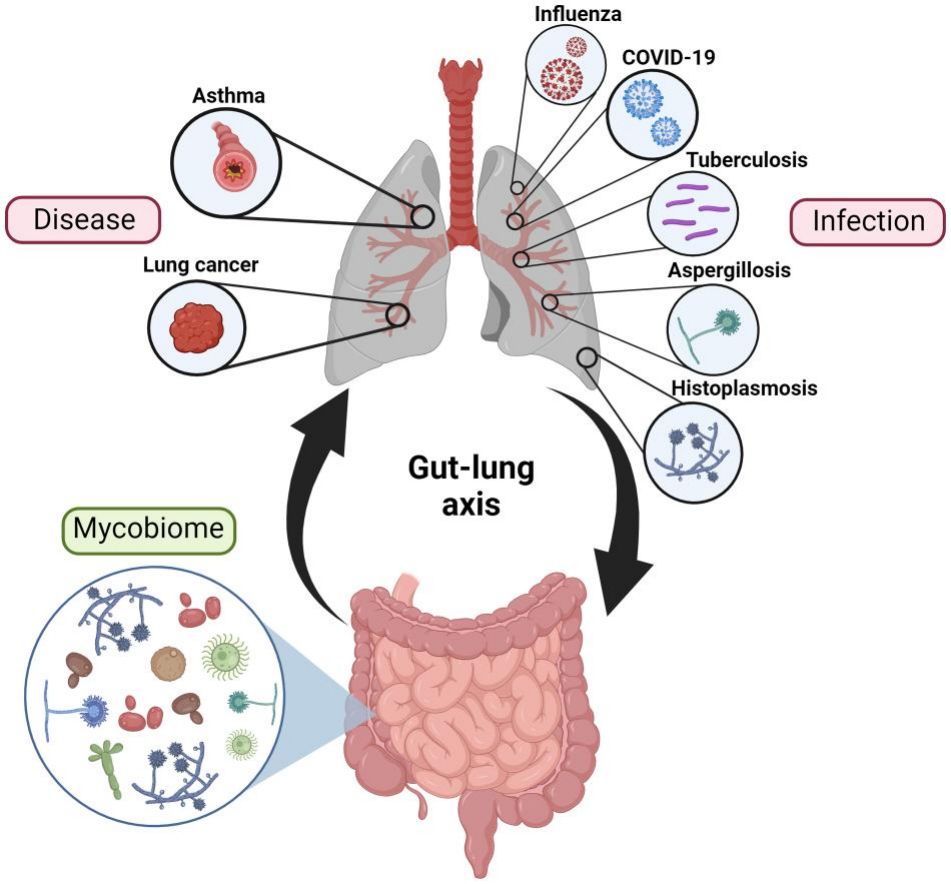
Gut mycobiome effects on allergic airway disease. Relative abundance of various fungal taxa can occur in the gut through various factors including oral antibiotics and antifungals. The relative abundance of specific fungi in the gut is linked to severity and susceptibility to allergic airway disease. Overgrowth of *Candida* species increases susceptibility to allergic airway disease induced by *A. fumigatus* and house dust mite (HDM) sensitization. In response to *A. fumigatus* sensitization, mice with overgrowth of *Candida* in the gut show increased eosinophils (E), mast cells (MC), and Th2 cells in the lung. Furthermore, in humans, Th17 cells have been isolated from asthma patients that show cross-reactivity against *C. albicans* and *A. fumigatus*. Sensitization with HDM in mice promotes influx of innate lymphoid cells 2 (ILC2s) which influence Th2 cells in the lung. Oral antifungals such as fluconazole increase abundance of *Aspergillus amstelodami*, *Epicoccum nigrum*, and *Wallemia sebi* which increase allergic airway disease susceptibility following HDM sensitization. The cells involved in this response are unknown. However, high abundance of *W. mellicola* in the gut induces Th2, Th17 and IgE responses in the lung following HDM induced sensitization in mice. In humans, infants with high abundance of *Candida* and *Rhodotorula* in the gut have increased risk of asthma in childhood. Created with BioRender.com

The presence of specific fungal species in the human gut in early life are associated with increased risk of with atopic asthma [11, 52, 53]. For instance, low *Malassezia* taxa and high *Candida* or *Rhodotorula* taxa in the gut mycobiome of neonates leads to an increased proportion of Th2 cells and an escalated risk of asthma development in childhood (Fig. 1) [11]. Two large studies, the Ecuador Life (ECUAVIDA) study and the CHILD Cohort study in Canada, demonstrated that an increased abundance of *Candida krusei* in neonates was correlated with an elevated risk of asthma in childhood [52, 53]. Importantly, the overgrowth of *C. krusei* was also associated with the early-life use of antimicrobials [52]. Additionally, a recent small pilot study in humans revealed that increased *C. albicans* in the gut is linked to severe asthma exacerbations [48]. Thus, abundance of various fungal species in early life, especially *Candida* spp appear to impact asthma development and severity in childhood, providing a potential prognostic biomarker for asthma development. However, it is important to note these studies may represent a correlation rather than causation.

*Candida albicans* is the most abundant commensal fungi in the human GI tract. Early mouse studies indicated that antibiotic administration or fungal colonization with *C. albicans* in the gut promotes the development of *A. fumigatus*-induced allergic airway disease [49] (Fig. 1). Moreover, these responses were associated with increased levels of eosinophils, mast cells, Th17 cells, and Th2 cells in the lung [49]. Notably, memory T cells isolated from humans that exhibit specificity towards *C. albicans* also demonstrate considerable cross-reactivity with the inhaled environmental fungus *A. fumigatus* (Fig. 1) [54]. These cross-reactive cells can be found in patients with asthma, but also other lung inflammatory disorders, including COPD, and CF [55]. In mice, intestinal colonization with *C. albicans* led to increased Th17 cells in the lung and was associated with an increased susceptibility to allergic airway disease [56]. Overall, these data suggest that commensal fungi in the gut, like *C. albicans*, prime cross-reactive T cells that are recruited to the lung during inflammatory airway diseases.

Although *Candida* is the most abundant fungus in the gut, abundance changes in other fungal species have been shown to also impact allergy-induced asthma responses. For example, colonization with *Wallemia mellicola* in Altered Schaedler Flora (ASF) mice, which harbour a defined community of eight bacterial species, demonstrated heightened allergic airway disease severity following HDM sensitization, compared to ASF mice without fungi [57]. The addition of fungi to these mice did not significantly impact the bacterial composition in the gut. The observed responses in the lungs were associated with amplified Th2 and Th17 responses via IL-13 and IL-17, respectively, and increased IgE production by B cells (Fig. 1) [50, 57, 58]. These data highlight the potential huge impact that can occur from low abundance microbial communities. Therefore, it is important to consider the impact of less abundance gut fungal commensals on subsequent lung immunity. Additionally, fungal commensals are known to expand in specific treatment conditions such as prolonged use of antibiotics [59].

Long-term use of antibiotics is known to affect the gut microbiota composition and the development of allergic airway disease [60]. For instance, early-life exposure to antibiotics escalates the risk of asthma development in childhood [44, 61]. The effect of long term antifungal use on asthma development has not been researched in detail, however, advances in transplant medicine, HIV management, and cancer therapies in recent years have increased the prolonged use of antifungals [62]. In mice, disruption of the gut mycobiota with oral administered antifungals like fluconazole, amphotericin B, and 5-fluorocytosine (5-FC) leads to exacerbated allergic airway disease, manifesting as increased eosinophils and Th2 cells in the lungs (Fig. 1). Interestingly, despite reduced *Candida* species as a result of the fluconazole treatment, other fungi such as *Aspergillus amstelodami*, *Epicoccum nigrum*, and *Wallemia sebi* are expanded. Treatment with antifungals in the absence of gut fungi did not influence allergic airway responses, and bacterial diversity was maintained [50, 58]. Therefore, authors linked the effects on allergic airway disease to altered abundance of the aforementioned fungal species. Additionally, the supplementation of mice with a mix of *A. amstelodami*, *E. nigrum*, and *W. sebi* increased allergic airway disease following HDM sensitization [50]. Thus, these findings suggest that fluconazole treatment heightens susceptibility to asthma in mice through the relative overgrowth of specific fluconazole-resistant fungal species.

Gut derived macrophages and ILC2s have been suggested as possible intermediates connecting gut fungi to lung immunity [50, 51]. There are various macrophage types in the gut, including CX3CR1 + macrophages which are crucial for early control of *Candida* by restricting caspase-dependent apoptosis and promoting Akt phosphorylation [51]. In humans, a polymorphism mutation of the CX3CR1 gene impairs IgG generation following recognition of fungi such as *C. albicans*, *Pichia kudrazevii*, *S. cerevisiae*, and *A. amstellodamii* [50]. Interestingly, in mice, specific depletion of CX3CR1 + mononuclear phagocytes (MNPs) in the gut reduced allergic airway inflammation following HDM sensitization. Moreover, the depletion of these cells or the inhibition of syk-dependent signalling in these cells diminished the increase in Th2 cells and eosinophils during lung allergic inflammation [50]. Hence, the recognition of gut fungi by CX3CR1 + macrophages may mediate asthma induced by HDM sensitization. However, although these gut resident macrophages have been linked to lung immunity the precise mechanisms by which they elicit their effects in the lung is unknown. Authors speculated that they may impact Th2 cells in the gut that translocate to the lung to cause hyperinflammation [50]. More recent studies in mice have shown that antibiotic-treatment, followed by colonization with *C. albicans* in the gut display increased ILC2s in the lungs, augmenting eosinophil and Th2 inflammation following HDM sensitization (Fig. 1) [48]. Collectively, the results of the above studies suggest that innate immune cells play a direct role in the development of asthma following recognition of gut fungi. Similar effects have been observed for gut bacteria, where changes in the gut bacterial microbiota facilitate the migration of ILC2s from the gut to the lungs [63].

Metabolites produced by fungi have been researched in less detail. The impact of SCFAs production by gut commensal A. fumigatus on health and disease has not been investigated [64]. *Candida* is known to produce PGE2 from arachidonic acid, to promote colonization within the gut and enhance survival in phagocytes. In mice, *Candida* overgrowth in the gut results in increased plasma concentrations of prostaglandin E_2_ (PGE_2_) that induce M2 macrophage polarization in the lung, exacerbating allergic airway inflammation [65]. Therefore, microbial-derived products in the gut may translocate to the lungs, influencing allergic airway disease.

### Lung cancer

Lung cancer is the leading cause of cancer related deaths and caused an estimated 1.8 million deaths worldwide in 2020 [66]. There were an estimated 2.2 million new cases of lung cancer diagnosed worldwide in 2020 [66]. In the past few years, the gut microbiome has been linked to diagnostic biomarkers, initiation of tumorigenesis, disease progression, and efficacy of treatments for lung cancer [67]. Furthermore, in mice and humans with lung cancer, there is an increase in the gut permeability, epithelial turnover and microbial diversity, independent of chemotherapy effects [68, 69]. Thus, deeper study of the gut microbiome may shed new light into the cancer field of the respiratory tract.

In the realm of microbiome research concerning lung cancer, the predominant focus has been directed towards the study of bacteria. Studies exploring the influence of gut bacteria have highlighted that an imbalance within Bacillota (Firmicutes) and Bacteroidota significantly escalates the susceptibility to lung cancer [70, 71]. Moreover, the diversity of bacterial microbiota has exhibited a positive correlation with the efficacy of immunotherapy [70]. There is limited research on the gut mycobiome, but initial studies have detected expansion of pathogenic fungi among lung cancer patients [72]. Furthermore, individuals diagnosed with cancer, including lung cancer, demonstrate an expansion of *Candida* in the gut, likely due to increased use of antibiotics, is associated with an increased predisposition to systemic candidiasis [72, 73].

Lung adenocarcinoma (LUAD), a subtype of non-small cell lung cancer (NSCLC), has been associated with increased fungal diversity and richness within the gut, a trend that intensifies as the disease progresses [72]. Consequently, researchers suggest a potential promotion of disease progression by gut fungi. However, the temporal sequence, whether disease progression precedes a change in fungal diversity or vice versa, necessitates further exploration. Notably, at the phylum level, the gut's composition shows a decrease in Ascomycota and a concurrent increase in Basidiomycota [72]. The most significant changes were observed for *Candida* and *Saccharomyces* spp, with a reduction in *Candida* spp and an increase in *Saccharomyces*, *Aspergillus*, and *Apiotrichum* spp [72]. These marked distinctions in the gut mycobiome of lung cancer patients have been identified, but the repercussions of this fungal composition on disease prognosis and immune cell function remain unexplored.

Patients afflicted with lung cancer, akin to other cancer types, have an elevated risk of secondary ailments and syndromes like cancer cachexia. Cancer cachexia (CC), a metabolic syndrome observed in various cancers, is typified by muscle mass reduction, body fat depletion, and chronic inflammation. In a murine model of CC, followed by induction of Lewis lung carcinoma (LLC), mice show significant differences in the gut mycobiota [74]. More specifically, an increased abundance of certain fungal species, including Sordariomycetes, Saccharomycetaceae and *Malassezia* spp was observed [74]. Intriguingly, specific fungi like *Rhizopus oryzae*, known for producing gallic acids capable of suppressing tumorigenesis and delaying inflammatory responses, exhibited decreased presence [75]. Authors suggest this could be a potential probiotic candidate in adjuvant therapies to prevent or treat CC [74]. Hence, the indirect repercussions of cancer-associated syndromes on gut fungi might significantly influence lung cancer progression and could therefore represent a promising novel target for probiotic treatment.

Cancer patients, including lung cancer patients face an increased risk of systemic candidiasis [73]. This is largely due to the effects of cytotoxic chemotherapy that lead to increased gut barrier permeability allowing translocation of microbial pathogens, and leukopenia. Furthermore, use of chemotherapeutic agents, alongside increased use of broad-spectrum antibiotics leads to reduced bacteria in the gut contributing to amplified presence of *Candida* and other fungi, increasing the risk to develop systemic candidiasis [73]. A recent study in lung cancer patients found that those with high levels of *Candida* in the gut also demonstrated elevated levels of *Lactobacillus* spp, known to impede the formation of *Candida* hyphae and encourage colonization [76]. Hence, the perturbation of the gut microbial balance might promote *Candida* overgrowth and subsequent systemic infection.

### Other lung diseases

Recently two separate studies have started to explore the effect of the gut mycobiota in less common CRDs: Idiopathic pulmonary fibrosis (IPF) and bronchiectasis. IPF is a progressive lung disease characterized by the thickening and stiffening of lung tissue. It affects around 3 million people worldwide and mainly occurs in those ages over 50 years, but the prognosis is poor with an average survival of 3–5 years [77, 78]. In mice, IPF is induced via bleomycin administration which promotes lung fibrosis. Overgrowth of *C. albicans* in the gut of these mice leads to exacerbated pulmonary fibrosis [79]. Authors found no alterations in macrophage polarization, Th1 or Th2 cells, but found increased Th17 cells in the lung and enhanced Il-17A production by these cells [79]. IL-17 is known to exacerbate pulmonary fibrosis through neutrophil recruitment, increased production of inflammatory cytokines, epithelial–mesenchymal transition and fibroblast activation. Thus, these data highlight an important role of gut fungi in IPF progression.

Bronchiectasis is a heterogeneous respiratory condition, with multiple endotypes and is usually a co-morbidity of other CRDs [80]. In general, the underlying pathophysiology involves inflammation, destruction of the airway structure, and the irreversible widening of the smaller airways [81]. A recent study of 57 patients with bronchiectasis showed that high gut levels of *Saccharomyces* correlated with increased exacerbations and higher severity of disease [82]. While patients with lower disease severity had a higher abundance of *Candida* in the gut compared to healthy controls [82]. Thus, changes in the gut mycobiome may impact disease severity in patients with bronchiectasis, however, further research is required to understand these findings.

## Gut mycobiome and respiratory tract infection

The impact of the gut mycobiota has been linked to the disease severity of several lung infections, including those caused by viruses such as influenza viruses and SARS-CoV-2, bacteria including *Mycobacterium*, and fungi such as *Aspergillus* and *Histoplasma*.

### Viral lung infections

Several studies have highlighted the impact of the gut microbiota on the production of type I interferons (IFNs) in the lung which are well known to control viral infections.

#### Influenza

Influenza instigates seasonal epidemics, resulting in up to 5 million severe cases and an estimated 400 000 fatalities annually worldwide [83]. In humans, the gut mycobiota composition is altered in individuals infected with the H1N1 strain, displaying reduced fungal diversity and the prevalence of specific taxa compared to healthy counterparts [84]. Notably, in influenza patients the presence of *Aspergillus* spp in the gut correlates positively with levels of the systemic inflammatory marker C-reactive protein, while the presence of *Mucoromycota* correlate negatively with C-reactive protein and procalcitonin [84]. This might indicate the potential and differential contribution of these fungi to the disease severity and outcome of patients with influenza.

Animal studies have further revealed the link between the gut mycobiome and progression of influenza. Mice treated with antibiotics show increased susceptibility to lethal influenza virus infection [85]. Interestingly, symbiotic fungi can functionally substitute the protective benefits of intestinal bacteria, offering protection against mucosal tissue damage and positively modulating the reactivity of circulating immune cells [86]. More specifically, colonization of the gut with either *C. albicans* or *S. cerevisiae* in antibiotic-treated mice effectively reversed susceptibility to Influenza A virus infection, resulting in reduced mortality rates and enhanced responses from virus-specific CD8 + T cells [86]. This protective ability of commensal fungi was facilitated by mannans, a highly conserved component of fungal cell walls that, when administered to mice, reduced disease susceptibility in mice lacking commensal bacteria [86]. These findings support the benefits of the main fungal gut colonizers in halting progression of influenza lung infection.

#### COVID-19

COVID-19, caused by the novel coronavirus SARS-CoV-2, emerged as a global pandemic with varying clinical presentations from asymptomatic cases to severe respiratory compromise and multi-organ failure [87]. Patients with COVID-19 exhibit altered gut microbial composition and dysfunction of the gut mucosa, potentially increasing the translocation of microbial products and toxins, exacerbating the systemic inflammatory response [88].

Numerous studies have revealed alterations in the mycobiota of COVID-19 patients, showing both reduced and increased diversity, depending on disease severity and dominance of specific fungal species [89–91]. Notably, higher composition of *Candida* species, particularly *C. albicans*, is observed in patients with COVID-19 compared to healthy controls [90]. Overall, these studies highlight a higher composition of *Candida* species in COVID-19 patients, with a dominance of a single fungal species being more pronounced in critically ill patients [90]. Authors suggest this could be due to antibiotic use in these patients, which is well known to promote overgrowth of *C. albicans* [92–94]. Alterations in the gut mycobiota were sustained for up to 6 months, however, by this time the mycobiota was beginning to recover with slightly increased diversity [89]. The lasting effect of the gut mycobiome alterations in COVID-19 patients may have a prolonged impact on immune health and homeostasis. However, the impact of these alterations long term, or in patients with long COVID-19 has not been explored.

Patients with long COVID, a syndrome associated with sustained levels of immune activation and inflammation after initial infection with SARS-CoV-2, may be affected by translocation of commensal microbes. Higher levels of β-glucan, a component of the fungal cell wall, were found in patients with long COVID, suggesting fungal translocation [95]. Interestingly, this was not observed for LPS binding protein (LBP), a bacterial plasma biomarker. However, authors did not confirm that the β-glucan was from fungal origin, and may have originated from some bacterial species, or food such as oats and barley [96]. Furthermore, gram-positive bacteria would not have been detected on the LBP test. Overall, further studies are required to confirm the translocation of commensal microbes in patients with long COVID. Patients with severe COVID-19 who had intestinal *Candida* overgrowth demonstrated elevated levels of *C. albicans* specific immunoglobulin G (IgG) antibodies and systemic neutrophilia [87]. Studies in mice colonized with *C. albicans* from these patients, followed by SARS-CoV-2 infection, showed increased lung neutrophilia and pulmonary NETosis, suggesting an additional impact of *C. albicans* on exacerbating the effects of SARS-CoV-2 infection in the lung [87]. Treatment with fluconazole or interleukin-6 receptor blockade in these mice reduced circulating neutrophils and inhibited NET formation in the lungs, hinting at potential mycobiota-immune therapeutic strategies [87].

### Bacterial infections

The impact of gut mycobiome composition during bacterial infections in the lung has not been researched extensively. Studies in GF mice or mice treated with antibiotics, show a decreased survival following pulmonary infection with *K. pneumoniae*, *S. aureus*, *S. pneumoniae* or *P. aeruginosa,* compared to SPF mice [97–99]. However, the contribution of gut fungi in these models requires further investigation. So far, the effect on gut fungi during bacterial lung infections, or vice versa has only been investigated in the context of tuberculosis (TB), and is discussed in the next paragraph.

#### Tuberculosis

Each year there are over 10 million new cases of tuberculosis (TB), and around 1.5 million deaths worldwide [100]. Treatment involves a combination of antibiotics over several months to eradicate the bacteria, with early diagnosis and treatment crucial for preventing the spread of the disease and reducing complications [101]. Recently, the gut mycobiota has been shown to be altered in TB patients, both on treatment and without treatment. However, mycobiota alterations were more pronounced in patients who received long-term anti-TB treatment. The *Nakaseomyces*, including pathogenic species such as *Nakaseomyces glabratus* (previously *Candida glabrata*) were enriched in TB patients and showed further enrichment following anti-TB treatment [102]. Additionally, *Purpureocillium lilacinum*, an emerging pathogenic species that can cause pulmonary, ocular, and cutaneous and/or subcutaneous infections was not observed in healthy controls but was significantly increased in the gut mycobiome in patients with TB [102]. Authors suggest this increase of pathogenic fungal species may be linked to the increased risk of fungal co-infections observed in patients with anti-TB treatment [103, 104]. Additionally, these changes in flora composition were observed for up to over a year, and may affect future treatments and overall homeostatic immune health [102].

### Fungal infections

#### Aspergillosis

Infections in the lungs caused by *Aspergillus* give rise to various conditions, spanning from mild allergic responses to severe invasive disease [105]. The pivotal role of intestinal microbiota in shaping the anti-*Aspergillus* immune response in the lungs has been shown in mice. During *A. fumigatus* infection in mice, administering antibiotics decreased the population of Th17 cells in the lungs, correlating with reduced colonization of segmented filamentous bacteria (SFB) in the intestines [106]. Investigating the link between commensal SFBs and this phenomenon, the authors demonstrated that SFBs contribute to the accumulation of Th17 cells in the lung by inducing an increase in IL-1. This was confirmed when mice receiving serum pre-incubated with an IL-1 antagonist showed reduced Th17 cell response in the lungs [106]. However, this study did not investigate the effects of altered immune response on disease severity. As mentioned previously, *C. albicans* is known to promote Th17 responses and may also be contributing to the accumulation of Th17 cells. However, the fungal element of the microbiome was not explored in this study.

Cross-protective immunity between *A. fumigatus* and the gut commensal fungus *C. albicans* has been observed in both mice and humans. Mice inoculated with *C. albicans* in the gastrointestinal tract were protected from subsequent infection after intranasal *A. fumigatus* instillation and the development of invasive pulmonary aspergillosis [107]. This cross-protection was mediated by Th1 immunity and relied on IFN-γ. Additionally, Th1 cells reactive against the Crf1/p41 epitope were accountable for this cross reactivity [107]. These Th1 cell clones could be expanded *in vitro* from human peripheral blood mononuclear cells, suggesting that *Candida* colonization might expand Th1 cells with cross-reactivity to *A. fumigatus*, offering protection against invasive lung infections [107]. Additionally, identification of a specific T cell receptor (TCR) epitope that is cross reactive to multiple fungal species is crucial for the development of future therapeutics and vaccines.

#### Histoplasmosis

Histoplasmosis is an endemic mycosis but with a wide global distribution, most commonly found in North America, Latin America, South-East Asia, West and Southern Africa, and some European countries including Spain and Italy [108–114]. Pulmonary infection follows the inhalation of *Histoplasma capsulatum* spores. Histoplasmosis affects around 500 000 each year, of which around 100 000 develop disseminated disease which is associated with a 50% mortality despite treatment [115]. Interesting findings that involve the gut-lung axis have been observed through studies that explore the specific immune cells involved in the lung during histoplasmosis infection. CD11b + CD103+ dendritic cells (DCs) are typically found in the intestines. However, CD11b + CD103+ DCs were isolated from the lung of *H. capsulatum*-infected mice following anti-TNFα treatment [116]. Furthermore, these DCs induced a higher percentage of Tregs than control DCs *in vitro*. These CD11b + CD103+ DCs were also shown to migrate from the gut to the lungs during *H. capsulatum* infection [116]. Anti-TNF treatment induced migration of CD11b + CD103+ DCs from the gut to the lungs, enhancing the generation of Tregs in *H. capsulatum*-infected mice [116]. Thus, TNF neutralization increases susceptibility to pulmonary *H. capsulatum* infection by promoting the gut-to-lung migration of DCs that enhance Treg development. Although this paper did not show direct correlation with the gut mycobiome, anti-TNFα therapy, which is often given to patients with inflammatory bowel disease (IBD) has been shown to induce changes in the relative abundance of fungi in the gut [117, 118]. For instance, Crohn’s disease (CD) patients receiving anti-TNFα treatment have higher abundance of Ascomycota, and lower abundance of Basidiomycota, while CD patients with no treatment had high abundance of Basidiomycota [118]. Furthermore, in a cohort of paediatric patients with IBD, those who did not respond to anti-TNFα therapy were associated with higher abundance of *C. albicans* in the GI tract compared to responders [117]. Therefore, the link between anti-TNF therapy on gut mycobiota and subsequent immunity in the lung warrants further investigation.

## Conclusion

Understanding the intricate relationship between the gut microbiota and respiratory health, particularly in the context of fungi, represents an evolving frontier in medical research. Despite remarkable progress in elucidating the role of the gut microbiome in health and disease, the contribution of fungi in the gut microbiome remains significantly underexplored. For the available mycobiome findings to be fully integrated into clinical practice, we want to highlight the critical need for a more comprehensive understanding of the impact of fungi and fungal components on health outcomes. Furthermore, although advances are being made in the context of CRD such as asthma and lung cancer, there are no studies currently published that explore the gut mycobiome in other CRD such as COPD and CF. This research is particularly relevant for CF, where up to 60% of people with CF are infected with *Aspergillus* in their airways [105]. Furthermore, the effects of the gut mycobiome on the severity and outcome of respiratory infections is lacking, with most data available for viral infections. Future studies on the effect of the microbiome on lung disease progression and immunity should also consider commensal fungal species.

Studies have started to unveil the significance of the gut microbiota on the severity of lung disease, and the existence of a gut-lung axis, notably in humans. Specific bacteria and certain fungi have been implicated in disease development and progression. These findings have been propelled by investigations using GF mouse models and antibiotic treatment regimens to explore the effects of microbiota on lung physiology and diseases. The utilization of GF models has provided overarching insights into the role of the microbiota in lung infections and diseases, further paving the way for examining the specific effects of individual microbes on lung immunity. However, the use of GF mice and antibiotic treated mice have several limitations. For instance, GF mice show impairment of many aspects of immune development [119]. Thus, the impact of colonizing these mice with defined microbiomes and/or specific fungi may not reflect what would be observed in immunocompetent mice. Colonization of mice with fungal species, such as *C. albicans* is only possible in the absence of other microbes and precludes assessing its role in a combined microbiome. While GF mice provide a useful tool for initial observations, the impact of single microbes in the presence of whole microbial communities cannot be determined. Although the use of antibiotics or antifungals to influence the composition of the microbiome overcomes some of the issues observed in GF mice, this strategy also has several weaknesses. For instance, the significant reduction in microbial load also leads to gross changes in immune cell composition in the gut and periphery, and organ morphology like those observed in GF mice [120]. Additionally, antimicrobial treatments will impact other microbial communities such as in the lung. The increasing emergence of antibiotic/antifungal resistance could result in specific outcomes related to overgrowth of specific resistant species. Another important consideration interpreting and translating observations from microbiome studies in mice to humans, is that the normal microbiota communities in mice differ significantly from the microbiota communities observed in humans [121, 122]. For instance, up to 70% of humans are thought to be colonized with *C. albicans* at any one time, and this fungus is the most predominant commensal fungi in humans [123]. However, in mice *C. albicans* is not a commensal organism. Instead, *C. tropicalis* is commonly found as a commensal organism in mice [124]. Due to the increase in interest of the effects of the microbiota on health and disease it is likely that these models will be used more regularly, and so consideration of these caveats is an important consideration for the field.

Several fungal species have emerged which show increased prevalence in the gut during lung disease, particularly *Candida* spp. However, most studies to date which investigate changes in microbiome composition in people with CRD compared to healthy controls may reflect a correlation rather than causation. Further studies are required to define the link between the presence of fungi and disease outcome. Additionally, these fungi appear to show differential effects depending on the type of lung disease. For instance, presence of *C. albicans* has been linked to increased incidence of asthma in humans but was associated with decreased severity of invasive aspergillosis in a mouse model [49, 107]. The impact of the gut microbiome on lung immunity is highly complex, and more detailed studies that unpick the immune cell regulation during health and disease are required to understand these responses.

Determining the impact of specific components within the microbiome which can be utilized to develop treatments regimes is on the research horizon. Probiotics have emerged as a potential therapeutic avenue in modulating the host’s intestinal microbiota to protect against pulmonary infections. Clinical studies, particularly in intensive care units, have explored probiotics for preventing and treating nosocomial pulmonary infections, indicating the exciting potential of microbiota modulation, especially with probiotic strains from the Lactobacilli [125]. These interventions have shown promising results, demonstrating enhanced phagocytic activity, reduced bacterial load in the lungs, decreased tissue inflammation, and an anti-inflammatory environment that supports resolution of disease. Exploring the impact of gut fungi could be used in similar probiotic strategies. In fact, fungi have emerged as a promising probiotic candidate due to their resilience to the acidic environment of the stomach, and, in the case of several *Saccharomyces* spp, are non-pathogenic, but are able to colonize the gut and outcompete several pathogenic bacteria [126, 127]. Several studies and clinical trials using *Saccharomyces* spp, have shown promising results for the treatment of diarrhoea, inflammatory bowel disease, vulvovaginal candidiasis, and acne [128–133]. While these trials are promising, the administration of live fungi known to cause invasive infections, such as *C. albicans,* might potentially result in an increased risk of invasive infections. Therefore, careful consideration needs to be made when determining the risk-reward outcome of fungal prebiotics or postbiotics, particularly in immunocompromised patients who are more susceptible to invasive fungal infections [123, 134].

Understanding the impact of fungal-derived substances, such as candidalysin and farnesol secreted by *Candida* species, may be pivotal in understanding their influence on immune responses and evasion mechanisms. Candidalysin, through NLRP3 inflammasome-dependent cytolysis, contributes to phagocytic clearance evasion, while farnesol acts as a vital virulence factor impacting the differentiation and expression of pro-inflammatory cytokines [135, 136]. Additionally, fungal-derived prostaglandins and oxylipins play a crucial role in modulating immune responses, indicating a multifaceted interplay between fungal substances and host immune function [137]. As these substances can cross the epithelial barriers in the gut and enter the bloodstream, they may be important mediators of immunity at distal sites such as the lung.

Overall, gut microbiome studies are emerging that link fungal taxa to lung disease. However, to confirm these studies are indicative of a causal relationship, further studies are required that unveil the immune responses. Studies to date have largely focused on the effects on the gut mycobiota on T cell immunity, due to the ability of T cells to differentiate into long-lived effector and central memory T cells which rapidly respond to subsequent microbial infections. Some research is now starting to explore the effects of the gut mycobiota on innate cell response such as ILCs and macrophages. However, our understanding of how the intestinal mycobiota influences various immune and non-immune cells remains limited. Key immune cell types, such as natural killer cells, mast cells, and DCs, along with their interaction with intestinal epithelial cells, need more comprehensive exploration to elucidate the full spectrum of influences exerted by the gut mycobiome. A deeper comprehension of the interplay between gut fungi and lung health is vital for advancing clinical interventions and shaping future therapeutic avenues in respiratory healthcare.

## Data Availability

Data sharing is not applicable as no new data has been presented here.
